# Self‐knotted feeding jejunostomy tube in an esophageal cancer patient: A case report and review of the literature

**DOI:** 10.1002/kjm2.12869

**Published:** 2024-06-11

**Authors:** Po‐Hsuan Wu, Pei‐Shan Weng

**Affiliations:** ^1^ Department of General Surgery Kaohsiung Medical University Hospital Kaohsiung Taiwan; ^2^ College of Medicine Kaohsiung Medical University Kaohsiung Taiwan; ^3^ Department of Clinical Education and Training Kaohsiung Medical University Hospital Kaohsiung Taiwan

In patients who are unable to eat by mouth or have insufficient oral intake, artificial nutrition refers to the supplementation of daily metabolic nourishment requirements.^1^ A feeding tube, such as a feeding jejunostomy or gastrostomy, could be surgically implanted into the gut to achieve the equivalent outcome. However, surgical site infections, abdominal distension, colic, and metabolic problems are the most typically reported complications.^2^

Jejunostomy complications have been documented in the literature in between 2% and 12% of events.^2^ A self‐knotting jejunostomy tube is relatively uncommon, but if detected, it might result in unexpected damage. Herein, we report a 57‐year‐old male with spontaneously knotted feeding jejunostomy that was identified and safely removed without any complication.

A 57‐year‐old male with esophageal cancer presented to the outpatient department with obstruction of feeding jejunostomy. Seven months before the presentation, he had received a diagnosis of dysphagia, which was related to tumor obstruction, and a surgery of feeding jejunostomy was performed. The patient had abdominal pain, and the jejunostomy tube blockage was suspected during feeding. An abdominal x‐ray was arranged to confirm the placement of the tube. The abdominal radiograph revealed knotting and kinking of the feeding jejunostomy tube (Figure 1A). The jejunostomy was drawn until the resistance was encountered, and the pulling force was increased slightly to remove the self‐knotted feeding tube without additional invasive procedures (Figure 1B). A new feeding tube was inserted subsequently and allowed feeds to pass through.

**FIGURE 1:**
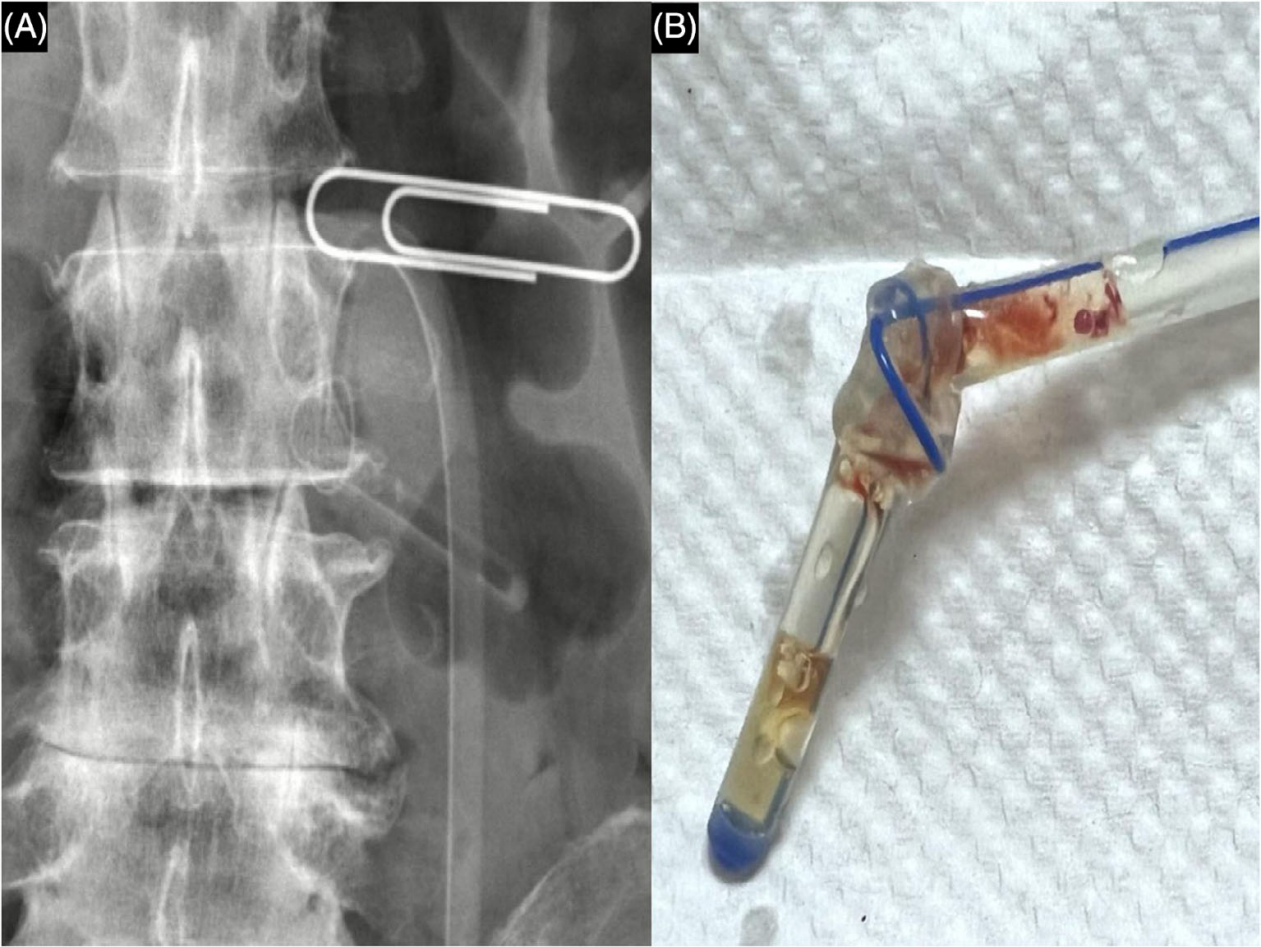
(A) The abdominal radiograph revealed knotting and kinking of the feeding jejunostomy tube. (B) Self‐knotted feeding jejunostomy tube was directly removed without additional invasive procedures.

Artificial feeding tubes could come with the additional risk of complications. Some are associated with the placement of the tube, such as small‐bowel obstruction, non‐obstructive small‐bowel narrowing, jejunal hematomas, and small‐bowel intussusception.^3^ Others are mechanical issues, such as coiling, kinking, occlusion, and dislocation.^2^ From the mechanical perspective, knotting appears to be a relatively unusual occurrence because the duodenum and jejunum's restricted area should prohibit motions that could cause the tube to knot.^4^ Fewer than 10 cases of such events in the last decade have been reported. Narrow diameter, excessive tube length, and prolonged duration of tube placement are factors that increase the risk of knotting.^5^ It has been proposed that intestinal dysmotility after surgery may cause the tubes to knot due to retrograde intestinal motility.^2^ While the exact cause of the knot remains unknown, its proximity to the tube hole site suggests that it may have formed due to the tube being forcefully inserted, creating a weak point that led to a loop and subsequent knot. In addition, the extra length of tubing may have allowed the jejunostomy tube to coil around itself, contributing to the formation of the knot. Previous reports indicate that the tube can become tangled after insertion, and the traction applied during recovery can tighten the knot.^4^

To remove the self‐knotted feeding jejunostomy safely, we could arrange an abdominal x‐ray to reveal the location of the knot. Once resistance was met, the jejunostomy tube was pulled with a continuous force to tighten and reduce the size of the knot. After that, we could use the Kelly clamp to adjust the depth of the feeding tube and to widen the opening of the stoma. Finally, the self‐knotted tube was held by Kelly's tip and pulled out smoothly.

Self‐knotted feeding jejunostomy could be prevented by employing broad bore tubes and proper lubricant.^5^ The placement process can be changed to move the feeding jejunostomy tube forward while flushing it with saline under gravity, avoid moving the tube forward when resistance is encountered, and shorten the tube's insertion length.^4^ The most reliable method to confirm the position is arranging plain film radiography after replacement.

## CONFLICT OF INTEREST STATEMENT

The authors declare no conflict of interest.
